# Data-driven models to predict shale wettability for CO_2_ sequestration applications

**DOI:** 10.1038/s41598-023-37327-2

**Published:** 2023-06-22

**Authors:** Ahmed Farid Ibrahim, Salaheldin Elkatatny

**Affiliations:** 1 Department of Petroleum Engineering and Geosciences, King Fahd University of Petroleum & Minerals, Dhahran, 31261 Saudi Arabia; 2 Center for Integrative Petroleum Research, King Fahd University of Petroleum & Minerals, Dhahran, 31261 Saudi Arabia

**Keywords:** Environmental impact, Carbon capture and storage, Scientific data

## Abstract

The significance of CO_2_ wetting behavior in shale formations has been emphasized in various CO_2_ sequestration applications. Traditional laboratory experimental techniques used to assess shale wettability are complex and time-consuming. To overcome these limitations, the study proposes the use of machine learning (ML); artificial neural networks (ANN), support vector machines (SVM), and adaptive neuro-fuzzy inference systems (ANFIS) tools to estimate the contact angle, a key indicator of shale wettability, providing a more efficient alternative to conventional laboratory methods. A dataset comprising various shale samples under different conditions was collected to predict shale-water-CO_2_ wettability by considering shale properties, operating pressure and temperature, and brine salinity. Pearson’s correlation coefficient (R) was utilized to assess the linearity between the contact angle (CA) value and other input parameters. Initial data analysis showed that the elements affecting the shale wettability are primarily reliant on the pressure and temperature at which it operates, the total organic content (TOC), and the mineral composition of the rock. Between the different ML models, the artificial neural network (ANN) model performed the best, achieving a training R^2^ of 0.99, testing R^2^ of 0.98 and a validation R^2^ of 0.96, with an RMSE below 5. The adaptive neuro-fuzzy inference system (ANFIS) model also accurately predicted the contact angle, obtaining a training R^2^ of 0.99, testing R^2^ of 0.97 and a validation R^2^ of 0.95. Conversely, the support vector machine (SVM) model displayed signs of overfitting, as it achieved R^2^ values of 0.99 in the training dataset, which decreased to 0.94 in the testing dataset, and 0.88 in the validation dataset. To avoid rerunning the ML models, an empirical correlation was developed based on the optimized weights and biases obtained from the ANN model to predict contact angle values using input parameters and the validation data set revealed R^2^ of 0.96. The parametric study showed that, among the factors influencing shale wettability at a constant TOC, pressure had the most significant impact, and the dependency of the contact angle on pressure increased when TOC values were high.

## Introduction

Various studies have highlighted the significance of the interaction between carbon dioxide (CO_2_) and shale formations in different applications related to CO_2_ sequestration^1–5^. One notable application involves the evaluation of enhanced oil and gas recovery techniques aimed at increasing hydrocarbon extraction from shale reservoirs. These techniques encompass approaches such as CO_2_ and N_2_ huff techniques, miscible gas injection, and CO_2_ flooding in shale oil reservoirs. Additionally, the research has explored the interaction between CO_2_ and shale formations in the context of carbon sequestration, where CO_2_ can be stored in various subsurface formations including depleted hydrocarbon reservoirs, saline aquifers, unmineable coalbeds, and oil reservoirs. Shale formations play a crucial role as cap rocks, effectively sealing and preventing CO_2_ leakage to upper formations. As injected CO_2_ migrates upwards due to its lower density compared to the formation brine, it becomes trapped by an ultralow seal cap rock.

The wettability of the shale/CO_2_ /brine system greatly influences the structural trapping capacity (of a caprock) and the cap rock integrity^4^. Where the more wet the shale rock, the more efficient the structural trapping, and higher cap rock integrity. Shale formations have also recently been considered to be CO_2_ storage. CO_2_ can be stored as a dissolved gas in the formation water, adsorbed phase in the shale matrix, or stored as free CO_2_ or supercritical fluid in the formations’ natural fractures and matrix pores. The adsorption storage capacity is more common in shale formations. This is explained by the fact that CO_2_ has a much higher capacity for adsorption than methane. The CO_2_ adsorption capacity in the shale matrix surface for CO_2_ sequestration in shale formation is determined by the wetting behavior of the shale in contact with CO_2_ in the presence of formation brine in the matrix. For example, in lower water wet formations, the CO_2_ diffusion rate to the rock surface will be greater than the diffusion rate through the hydrated layer on the rock surface in higher water wet formations^6–8^. Furthermore, for hydraulic fracturing operations, the water flowback efficiency is heavily influenced by spontaneous water imbibition and rock wettability^3^. Studying the effects of using CO_2_ and CO_2_ foam for drilling and hydraulic fracturing operations is another application of CO_2_ interaction with shale formations^1–6,9–12^.

Shale wettability can be assessed using various experimental quantitative and qualitative techniques. Contact angle measurements, the Amott method, the USBM method, and nuclear magnetic resonance (NMR) are an example of the quantitative methods^13–24^. In addition, there are other qualitative methods to measure rock wettability, such as flotation, relative permeability, and recovery curves. These methods have several limitations that can affect the accuracy and reliability of the predictions.

The contact angle measurements on shale surfaces are widely used but have limitations that are related to the complex surface preparation process. One limitation is that the test requires a clean and smooth shale surface, which can be difficult to obtain in practice. Additionally, the test can be affected by the presence of impurities or coatings on the shale surface, which can alter the CA-value and lead to inaccurate predictions. Furthermore, the test can be difficult to perform on shale samples with irregular surfaces, which can lead to inaccurate measurements and unreliable predictions.

### Machine learning applications

Machine learning (ML) can be used to analyze large and complex datasets to improve decision-making and automate tasks in the industry. ML has been used in various applications in the oil and gas industry such as seismic surveys, well logs, drilling parameters, and production data to create detailed models of reservoirs^25–29^.

Machine learning algorithms have gained significant traction in CO_2_ sequestration for reservoir characterization and management. They leverage various data sources to predict vital reservoir parameters such as porosity, permeability, and lithology, enabling accurate estimation of storage capacity and understanding of CO_2_ behavior. Furthermore, machine learning techniques enhance reservoir simulation models by incorporating real-time sensor data and dynamic reservoir activity, resulting in improved management, risk assessment, and precise estimation of CO_2_ transport and potential leakage risks. In rock wettability prediction, machine learning methods have been utilized. Wang et al. employed deep learning with nuclear magnetic resonance (NMR) for wettability estimation^30^, while Otchere et al. proposed an NMR-based approach for rock wettability prediction^31^. However, these methods often involve complex analysis and assumptions, introducing uncertainties. Moreover, they have not specifically addressed CO_2_ wettability in shale formations. Other studies have explored machine learning for contact angle measurement, such as^32^ analyzing droplet images and Ibrahim predicting wettability of coal formations. Tariq et al. focused on specific rocks using neural networks for rock wettability prediction with CO_2_, considering pressure and temperature as operating conditions^33^.

**Figure 1:**
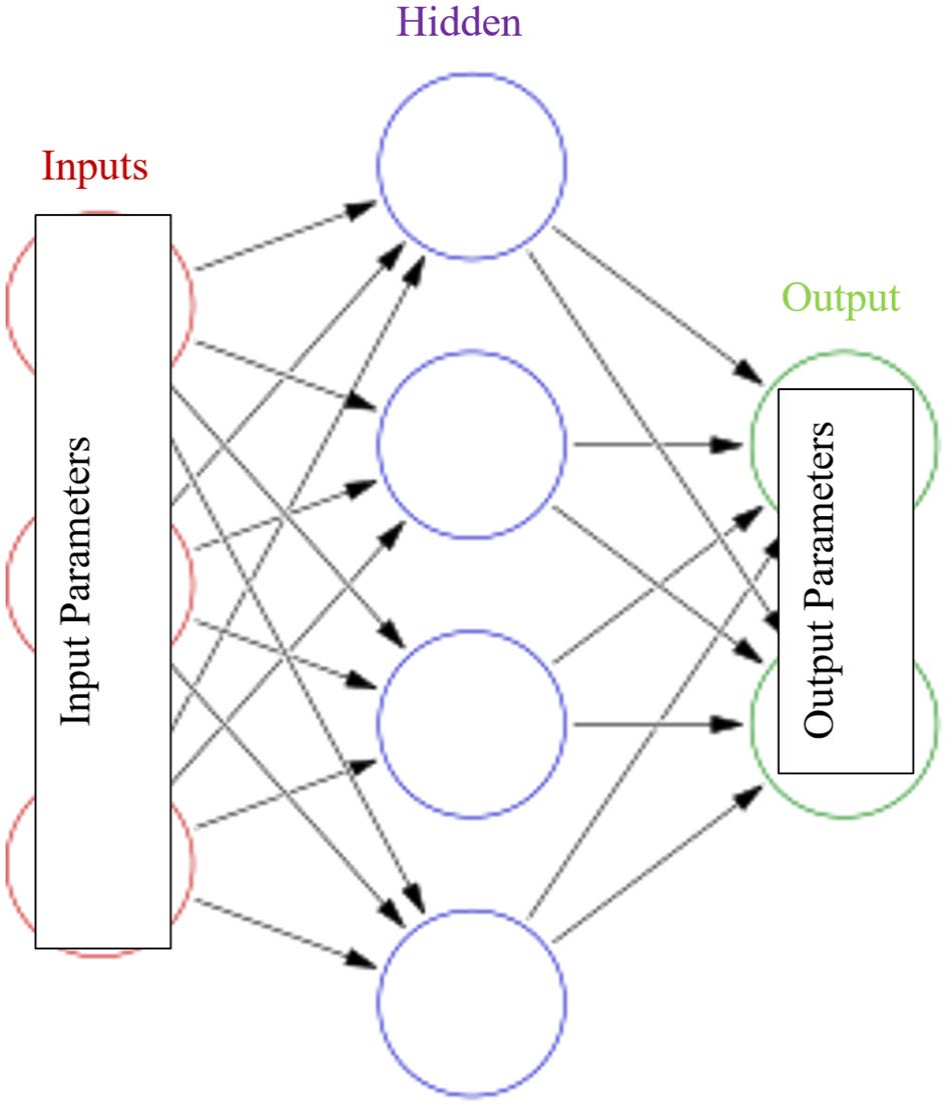
ANN structured of different hidden layers in addition to the input and output layers.

Artificial neural networks (ANN) is a popular machine-learning method that simulates the brain neurons. In classification, regression, and clustering tasks, ANN could be used as an unsupervised or supervised machine learning tool. As shown in Fig. 1, an ANN is made up of several elements such as neurons, training functions, and transfer functions in different layers. Many effective applications of ANN in the oil and gas industry have been reported in the literature^34–38^.

Support Vector Machine (SVM) is a powerful and widely used supervised learning algorithm for classification and regression tasks. The SVM algorithm seeks to find a boundary or a hyperplane that maximally separates the different classes in the data. The boundary or hyperplane is chosen so that it maximizes the margin, or the distance between the boundary and the closest data points from each class, known as support vectors. SVMs can handle both linear and nonlinear data by using kernel functions, which transform the input data into a higher dimensional space, where a linear boundary can separate the classes. Some popular kernel functions include the radial basis function (RBF) and the polynomial kernel. One of the main advantages of SVMs is that they are able to handle high-dimensional data and have a regularization parameter, which helps to avoid overfitting. Additionally, SVMs are able to handle data sets with a large number of features. SVMs are widely used in various fields such as bioinformatics, natural language processing, computer vision, and finance. SVM has different applications in oil and gas industry for classification and regression problems^38–40^.

Adaptive Neuro-Fuzzy Inference System (ANFIS) is a type of artificial intelligence system that combines the benefits of both fuzzy logic and neural networks. ANFIS was introduced by Jang in 1993 as a way to improve the performance of fuzzy inference systems by using neural network techniques for parameter estimation. ANFIS is widely used in various fields, including control systems, financial analysis, pattern recognition, and many other applications. ANFIS uses a hybrid learning algorithm that combines the benefits of both gradient descent and backpropagation. The gradient descent method is used to optimize the parameters of the fuzzy sets, while backpropagation is used to optimize the parameters of the neural network. ANFIS has several advantages over other types of artificial intelligence systems. One of the main advantages is that ANFIS can handle complex non-linear relationships between inputs and outputs, making it useful for many applications where traditional statistical models fail to produce accurate predictions. ANFIS is also easy to implement and can be trained using a variety of optimization techniques, including genetic algorithms and particle swarm optimization.

The importance of understanding shale wettability when exposed to CO_2_ lies in its implications for assessing the feasibility and effectiveness of CO_2_ sequestration techniques. The novelty of the current research is to introduce a novel approach to predict CO_2_ shale wettability using machine learning techniques to overcome the limitations of traditional experimental methods, which are time-consuming and resource intensive. By harnessing the power of machine learning, the study seeks to develop accurate and user-friendly models for estimating shale wettability based on the contact angle. Previous ML studies have either focused on different rock types, involved complex interpretations of input features, relied on a single machine learning method, or suffered from lower accuracy and overfitting problems. In contrast, this research utilizes advanced machine learning computational techniques to uncover meaningful patterns and correlations from different datasets. The current study comparing different ML methods including artificial neural networks (ANN), support vector machines (SVM), and adaptive neuro fuzzy inference system (ANFIS). Moreover, to eliminate the need for re-executing the machine learning models, an empirical correlation was formulated using the optimized weights and biases derived from the ANN model. This correlation enables the prediction of contact angle values by utilizing input parameters without the requirement of rerunning the machine learning models.

## Methodology

### Data description

This study utilized a compiled dataset of contact angle measurements from various literature sources that were carefully filtered to include only relevant data. To account for the impact of different rocks and conditions, input parameters such as rock mineralogy, total organic carbon (TOC), porosity, permeability (k), pressure (P), and temperature (T) were considered. The dataset was split into training and testing sets at a 70/30 ratio and used to train various machine learning models, whose results were validated on an unseen dataset.

**Figure 2:**
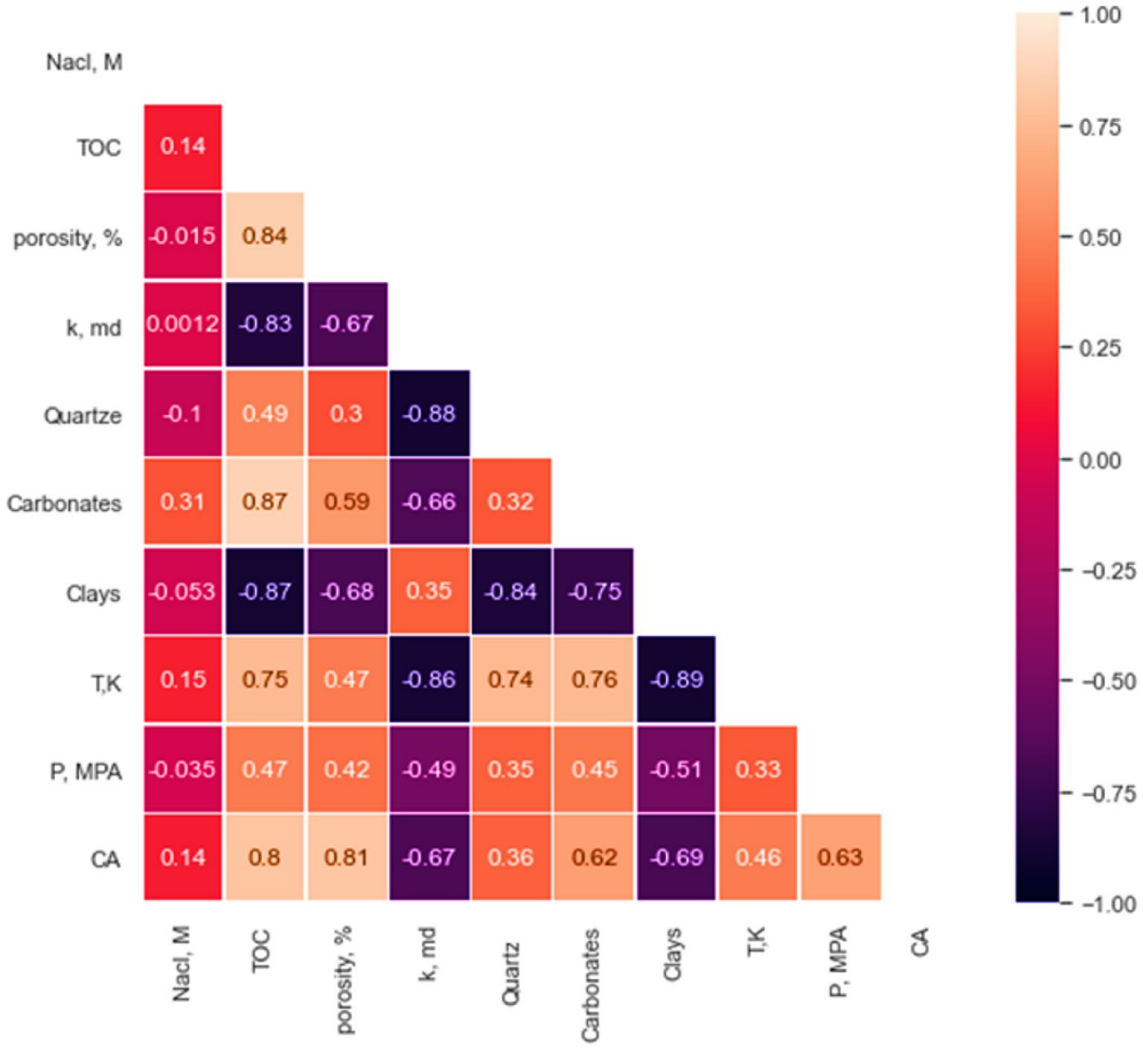
R-values between the different parameters with each other.

**Figure 3:**
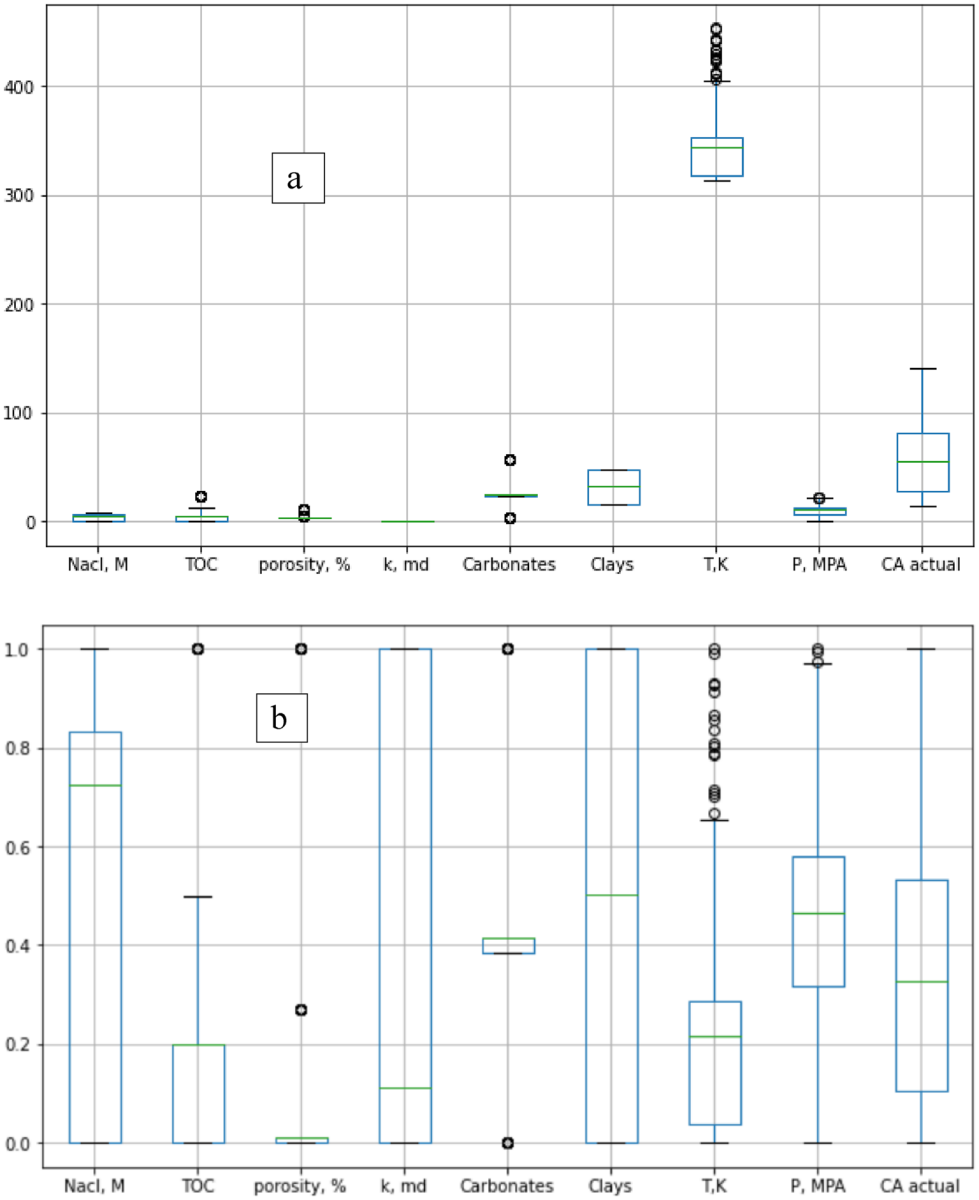
Box plot for different parameters (**a**) the actual data and (**b**) the min–max normalized data.

Table 1 presents the statistical parameters for different shale properties and operating conditions, along with their corresponding CA-values ranging from 14 to 140 degrees, indicating a broad spectrum of wettability from strong water wet to CO_2_ wet conditions. Figure 2 displays the correlation coefficient heatmap for the different inputs and outputs, showing the bivariate analysis of their relationship. Figure 2 quantifies the relationship between the properties with the R-value, where values range from − 1 for a strong negative relationship to + 1 for a strong positive relationship. The input parameters with the most significant impact on shale wettability were TOC, porosity, and clay contents, while CA-value showed the least sensitivity to quartz content and salt concentration in the brine. Figure 3 showed box plot for the different input and output data. Figure 3a showed the box plot for the actual values for the input and the output data. The input parameters showed various ranges. Figure 3b showed the normalized parameters level. Minimum–Maximum normalization technique was used to normalize the data to be in the same level and varies from 0 to 1. The data showed that some data are follows normally distribution such as clays, carbonate contents, pressure, and the contact angle values. On the other hand, TOC, and permeability follows lognormal distribution.

**Table 1:** Univariate analysis for the different parameters used in the study including rock characteristics, operating conditions and corresponding contact angle.

	Nacl, M	TOC	Porosity, %	k, md	Quartz, wt%	Carbonates, wt%	Clays, wt%	T, K	P, MPA	CA
MIN	0	0.1	2.8	7.75E−07	12	3.2	16	313	0	14.1
MEAN	3.9	4.1	3.6	6.24E−05	42.1	25.5	31.8	346.9	10.1	58.1
Median	5.1	4.8	2.8	1.76E−05	47.2	25	32	343	10	53.9
Standard deviation	2.6	5.6	2.1	7.18E−05	16.8	11.2	14.9	36.5	5.1	32.8
Coefficient of variation	0.67	1.37	0.58	1.15	0.40	0.44	0.47	0.11	0.50	0.56
MAX	7	23.4	10	1.52E−04	59	56	47.9	453	21.4	141.7

### Model development

**Figure 4:**
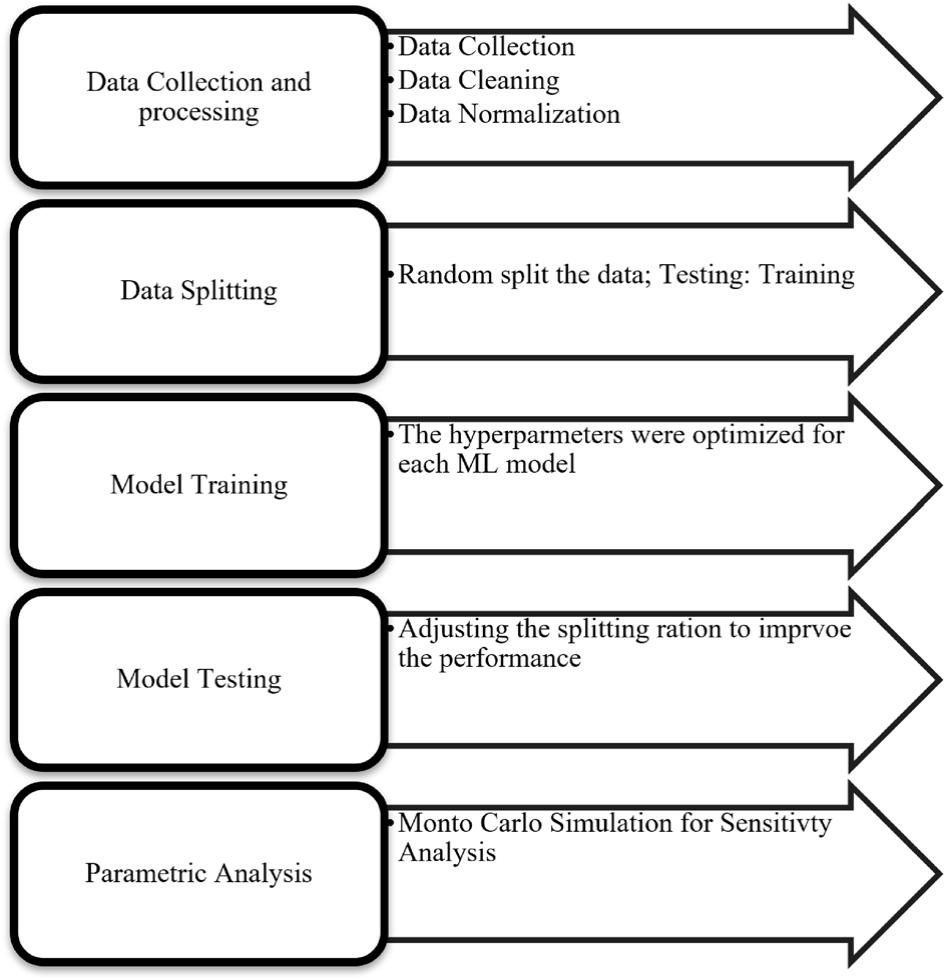
Models’ development processes.

In Fig. 4, the methodology employed to construct the ML models to forecast shale CA-value based on shale mineralogy and operating conditions is depicted. Initially, data was collected and preprocessed, followed by the utilization of various ML algorithms to predict the CA-value by incorporating shale properties such as permeability and porosity, mineralogy (including clay content, carbonates, and quartz), and total organic carbon (TOC), as well as input parameters like brine salinity, temperature, and pressure. Subsequently, the data was randomly divided into training and testing datasets, and the models were trained using the training dataset, with hyperparameters optimized to enhance performance. To assess the splitting ratio, the developed models were tested on the testing datasets, and a hidden dataset was utilized to validate the model.

Artificial neural networks (ANN), support vector machines (SVM), and adaptive neuro fuzzy inference system (ANFIS) were applied on the collected data. For each model different hyper parameters were optimized to reach the highest model performance. Table 2 summarizes the different hyperparameters options and the optimum option used on each ML model.

**Table 2:** Different hyperparameters options and the optimum selected option for different ML models.

Parameter	Available options	Optimum option
ANN model		
Number of hidden layers	1–3	Single hidden layer
Number of neurons in each layer	5–40	10
Training/testing split ratio	70–90%	(Training/testing) 70/30%
Training algorithms	Trainlm, trainbfg, trainrp, trainscg, trainbr, traincgf, traincgp, trainoss, traingdx	Trainbr
Transfer function	Tansig, logsig, elliotsig, radbas, hardlim, satlin	Logsig
Learning rate	0.01–0.9	0.05
ANFIS		
Number of membership function	2–10	5
Type of input membership function	Gaussmf, ‘linear’, trimf, gauss2mf, pimf	‘Gaussmf’
Type of output membership function	Gaussmf, ‘linear’, trimf, gauss2mf, pimf	‘Linear’
Cluster radius	0.1–5	0.5
Epoch size	5–500	100
Fuzzy network	genfis2, genfis1	genfis2
SVM		
Lambda	0.001 to 1	0.1
Epsilon	1E−5 to 1E−1	0.0001
Kerneloption	1–10	3.5
Regularization parameter	10–1000	200
Kernel	Gaussian, polynomial, Polyhomog, Htrbf, and Rbf	Gaussian

R^2^ and root mean square error (RMSE) were used to evaluate the developed ML models. R^2^ and RMSE were calculated using the following equations.

1 $${\mathrm{R}}^{2}=1-\frac{{\mathrm{SS}}_{\mathrm{E}}}{{\mathrm{SS}}_{\mathrm{YY}}},$$

2 $${\mathrm{SS}}_{\mathrm{E}}=\sum \limits_{\mathrm{i}=1}^{\mathrm{n}}{\left({\mathrm{y}}_{\mathrm{i}}-{\widehat{\mathrm{y}}}_{\mathrm{i}}\right)}^{2},$$

3 $${\mathrm{S}}_{\mathrm{YY}}=\sum \limits_{\mathrm{i}=1}^{\mathrm{n}}{\left({\mathrm{y}}_{\mathrm{i}}-\overline{\mathrm{y} }\right)}^{2},$$

4 $$\mathrm{RMSE}=\sqrt{\frac{{\mathrm{SS}}_{\mathrm{E}}}{\mathrm{n}}},$$

where SS_E_ is the summation of residuals squares, n is the number of data, ${y}_{i}$ is the actual CA-values, ${\widehat{y}}_{i}$ is the predicted CA-values, and ${SS}_{YY}$ is the summation of squares of data variation with respect to the data mean value.

## Results and discussion

### ANN model results

The artificial neural networks (ANN) model was created to estimate the contact angle (CA) based on the input parameters. Different hyperparameters were tested for the ANN model and the optimum performance was found using one hidden layer with 10 neutrons, the training function was selected to be “trainbr” and the transfer function is “logsig”.

**Figure 5:**
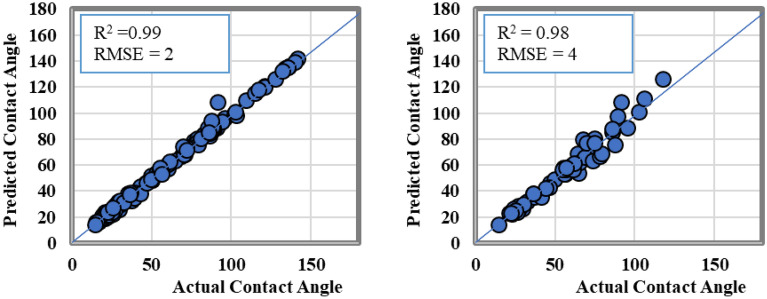
The actual versus the predicted CA-value from ANN model results.

Cross plots for the training and testing datasets of the ANN model are illustrated in Fig. 5. The results showed that ANN model was able to predict the CA-value using input parameters where the R^2^ values were higher than 0.98 for both the training and testing datasets, respectively. The predicted versus the actual contact angle values were aligned with the 45-degree line with RMSE value less than 4.

**Figure 6:**
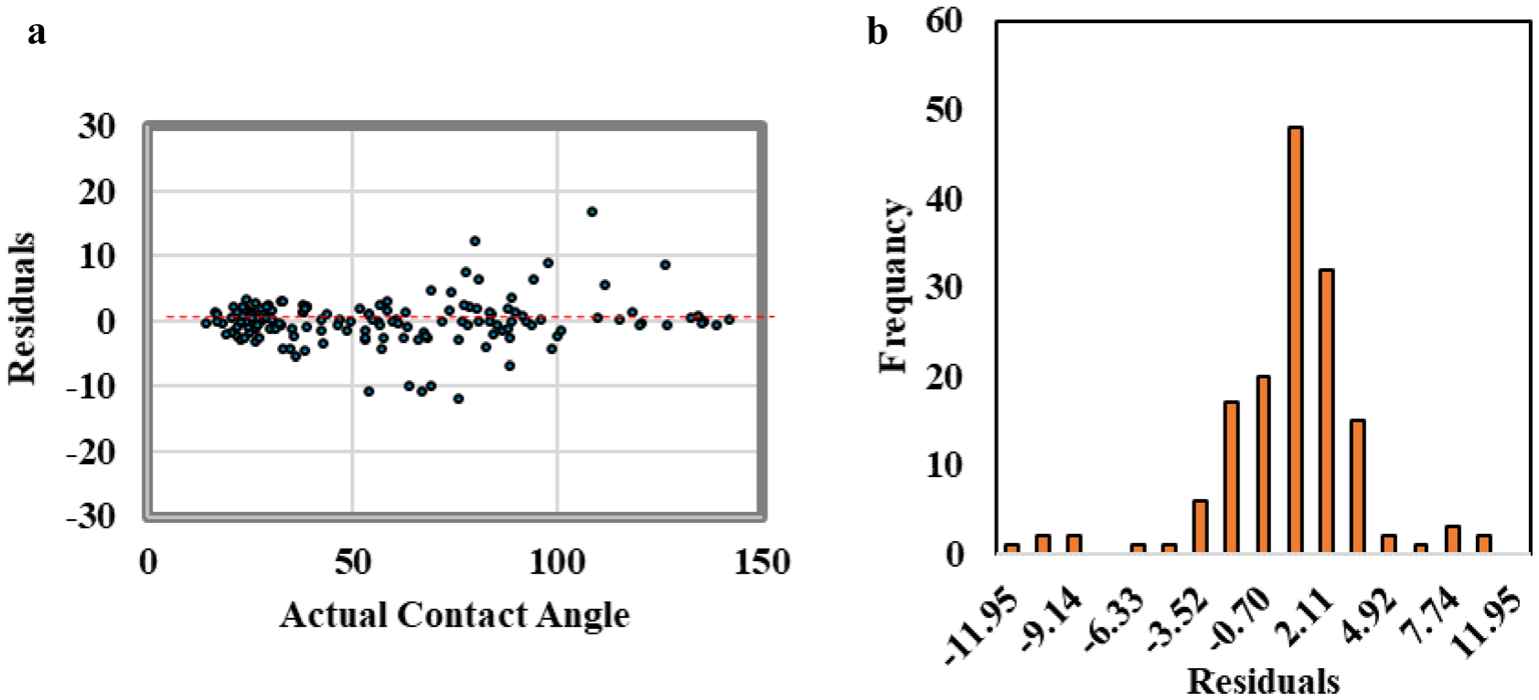
The residual error analysis, (**a**) scattered plot for the residual versus contact angle value, (**b**) residual frequency distribution.

The residual error analysis technique was used to analyze the residual between the actual and the ANN-predicted contact angle values. Figure 6a showed a scatter plot for the residual versus the contact angle values. The residual showed even scattering in positive and negative values along the contact angle values. In addition, Fig. 6b shows the frequency distribution of the residual values. The residual shows a normal distribution with mean value equal to zero that reflect good regression process without model biasing toward any contact angle ends.

The study produced new empirical equations that can be utilized to estimate contact angle without running the ANN code. These equations were derived from the weights and biases of the optimized ANN model^41–43^. The ANN model was built with a single hidden layer and logsig transfer function. Equation (5) details the resulting equation generated from these weights and biases.

5 $$CA=\left[\sum_{i=1}^{N=10}{W}_{2i} logsig\left(u\right)\right]+{b}_{2},$$

where $$u=\sum_{j}^{m}{W}_{1i,j}{X}_{j}+b{1}_{i}$$, and

$$logsig\left(u\right)=\frac{1}{1+{e}^{-u}}.$$

W_2i_ and b_2_ are the weight and bias between the hidden layer and output layer, ${W}_{1i,j}$ represents the weights at different neurons (i from 1 to N = 1) between the input layer and hidden layer for the different inputs’ parameters (j from 1 to m) including, NaCl molar concentration, TOC percentage, porosity percentage, k in md, quartz, carbonates, and clays concentrations, and operating temperature and pressure. b_1i_ denoting the optimized biases for the hidden layer neurons (i) from 1 to the total number of neurons (N). This equation was formulated to replicate the ANN-based model by utilizing the optimized networks' weights and biases. To substitute the weights and biases in Eq. (5), Table 3 lists the optimized weights and biases of the developed CA model.

**Table 3:** The optimized weights and biases of the developed ANN-based model.

i	b2	b_1i_	W1_i,j_ for different parameters									W2_i_
1	− 0.240	− 0.013	2.782	0.593	− 0.283	− 0.143	0.273	0.661	− 1.147	− 0.601	− 2.310	1.613
2		1.195	− 0.272	− 0.015	− 0.601	− 0.386	0.219	− 0.637	0.637	2.894	0.549	2.684
3		− 0.591	0.864	− 1.691	0.040	− 0.248	0.451	− 0.126	− 0.534	0.394	− 1.096	− 2.149
4		− 0.470	− 0.382	0.099	0.485	− 0.941	0.556	− 1.004	0.277	1.671	− 4.399	2.045
5		− 0.490	0.953	1.449	0.156	− 0.500	0.495	− 0.413	− 0.275	− 0.637	− 1.503	− 2.140
6		− 0.724	0.316	− 0.515	− 0.013	0.862	− 0.188	0.073	0.182	1.646	1.844	− 1.975
7		− 0.002	0.186	− 0.084	− 0.032	− 0.180	0.146	0.020	− 0.232	− 0.021	0.017	− 0.479
8		0.228	− 0.545	− 0.621	0.621	− 0.626	− 0.248	0.054	0.140	3.132	− 3.283	− 2.470
9		− 0.372	2.506	1.011	0.950	− 0.488	− 0.333	0.274	− 0.100	0.281	1.935	1.974
10		− 0.002	0.187	− 0.085	− 0.032	− 0.181	0.146	0.020	− 0.233	− 0.021	0.017	− 0.482

### ANFIS model results

Using the available data, an ANFIS model was constructed and subsequently trained and tested. The optimized hyperparameters for the ANFIS model included “Gaussian” and “Linear” for the input and output membership functions, respectively, with a membership function count of 5. Table 2 summarized the various options and the selected optimum hyperparameters.

**Figure 7:**
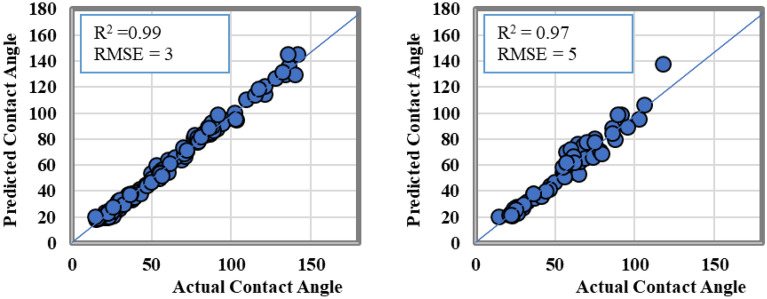
The actual versus the predicted CA-value from ANFIS model results.

To assess the accuracy of the ANFIS model, an across plot was generated and is shown in Fig. 7. The plot depicts the predicted values versus the actual values for the CA-value in both the training and testing datasets. The data scattered closely along the 45-degree line demonstrates the high accuracy of the ANFIS model’s predictions. The R^2^ values obtained were 0.99 and 0.97 for the training and testing datasets, respectively, providing additional evidence of the ANFIS model's ability to forecast shale CA-value based on rock properties and operational conditions. Furthermore, the RMSE values for both the training and testing datasets were below 5, which confirms the ANFIS model’s reliability.

**Figure 8:**
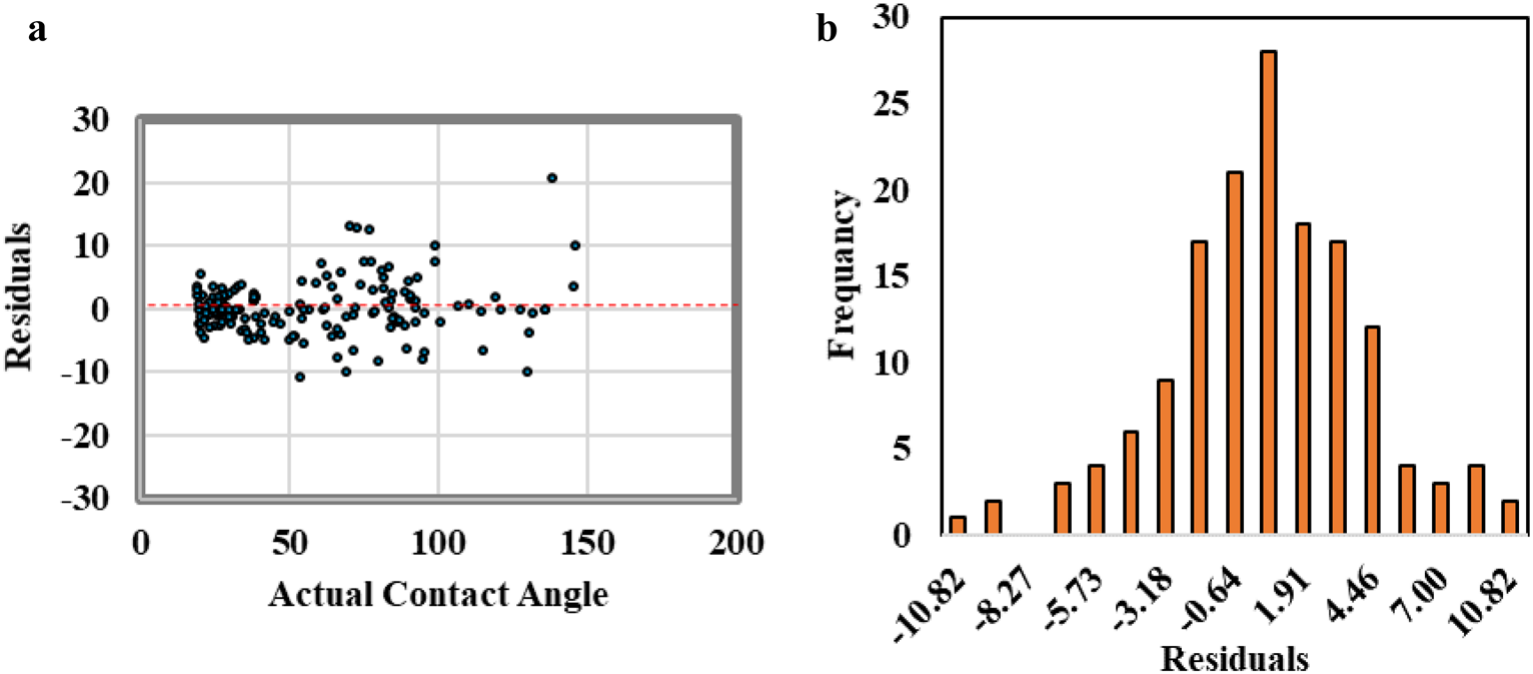
The residual error analysis for ANFIS model results, (**a**) scattered plot for the residual versus contact angle value, (**b**) residual frequency distribution.

Figure 8 shows that the residual error analysis of the predicted contact angle values from the ANFIS model exhibited normally distributed errors centered around a mean of zero. The residual values extended to − 10 and 10 degrees, which is indicative of the ANFIS model’s high accuracy in predicting CA values.

### SVM model results

Support vector machine techniques was also applied on the collected data to predict the contact angle values. As shown in Table 2, different hyperparameters were tested to improve the model performance. The optimum performance was found with using Gaussian as a kernel function with optimal kernel option = 3.5, epsilon = 0.0001, lambda = 0, and regularization parameter of 200.

**Figure 9:**
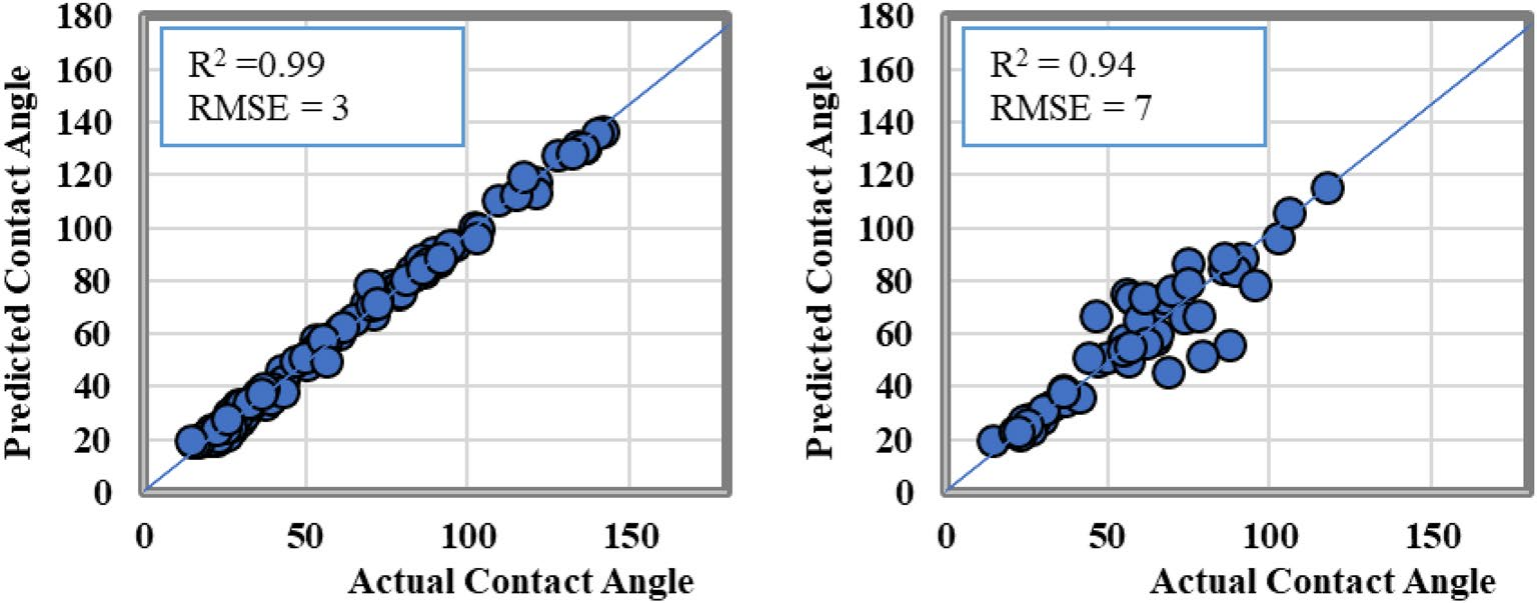
The actual versus the predicted CA-value from SVM model results.

Figure 9 displays cross plots of the training and testing datasets for SVM model. The SVM model showed an excellent predictive capability for CA values as a function of rock mineralogy and operating conditions. R^2^ values was found to be of 0.99 and 0.94 for the training and testing datasets, respectively, with RMSE values of 3 and 7 degrees in both datasets, which confirms its ability to predict the shale CA-value. This behavior may show some overfitting problem with SVM model where the training data set R^2^ was higher than the testing R^2^ value.

**Figure 10:**
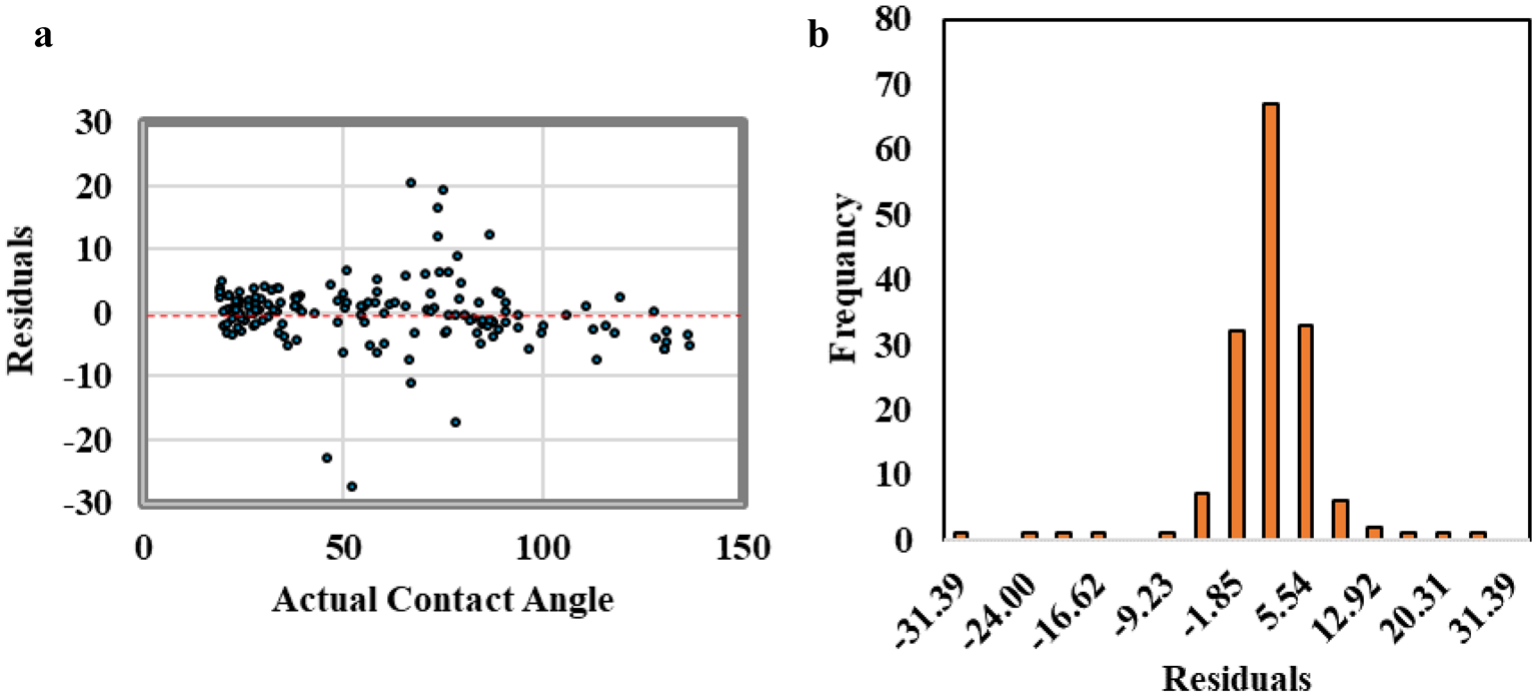
The residual error analysis for SVM model, (**a**) scattered plot for the residual versus contact angle value, (**b**) residual frequency distribution.

The residual error analysis of the predicted contact angle values from SVM model showed normally distributed error around mean of zero as presented in Fig. 10. Some residual values were extended to − 31 and 31 degree that increases the RMSE to 7 degree and the R^2^ becomes 0.94 for the testing data set.

### Models validation

**Figure 11:**
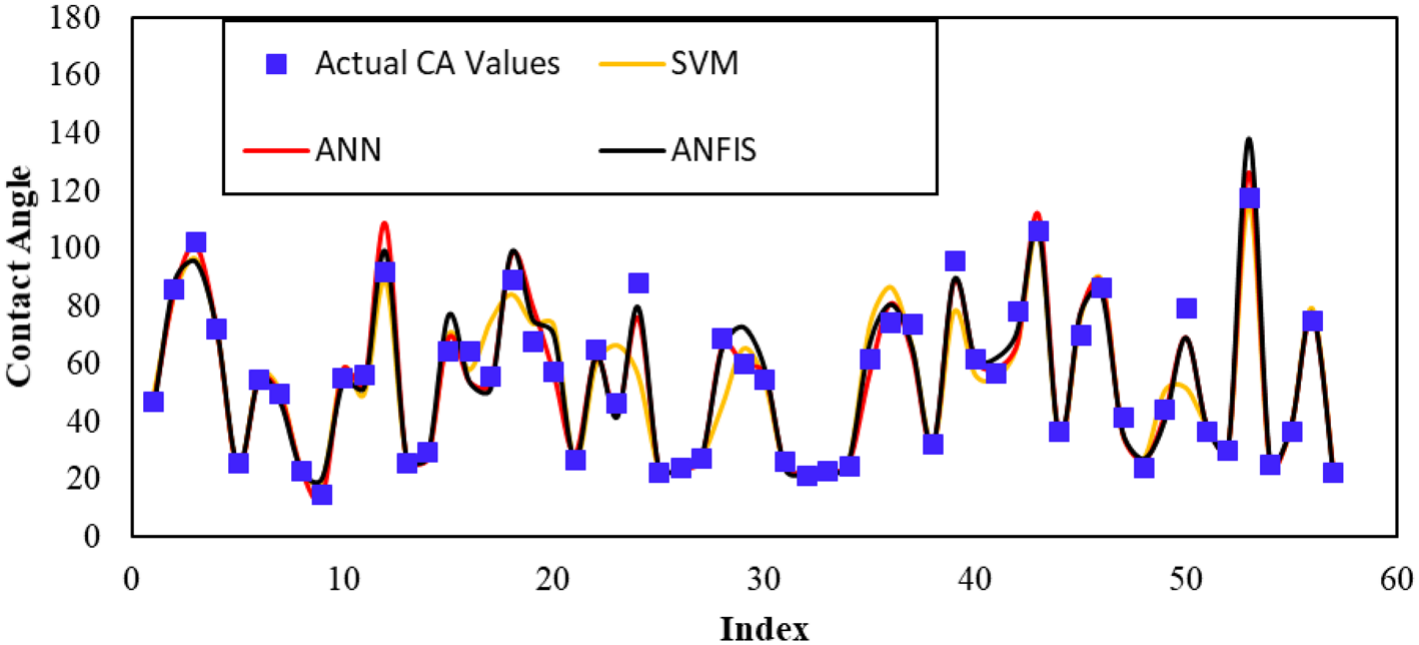
Actual versus the predicted parameters in the validation dataset for the different ML models.

Following the development of the ML models, they were validated using an unseen dataset. Figure 11 depicts the actual CA-value measurements compared to the predicted values for the different ML models. The actual CA-values are represented by dots, while the predicted values for the ML models are represented by various lines. As shown in Fig. 11, the ML models were successful in predicting shale CA-value based on formation properties and operating conditions. However, the SVM model failed to predict some of the CA-value in the validation dataset, with its results (yellow line) either overestimating or underestimating the actual values. In contrast, the ANN model demonstrated the best performance among the other techniques, achieving an R^2^ value of 0.96 in the validation dataset, with an RMSE of 5.

Table 4 summarizes the R^2^ and RMSE values for the different ML models, using various datasets. The results indicate that the ANN ML model’s performance was superior to the other models, followed by ANFIS and then SVM. The R^2^ for the SVM model was 0.88, indicating the presence of an overfitting problem that was observed in the testing set.

**Table 4:** R^2^ and RMSE summary of the different ML model’s prediction.

	R^2^			RMSE		
	Training	Testing	Validation	Training	Testing	Validation
ANN	0.99	0.98	0.96	2	4	5
ANFIS	0.99	0.97	0.95	3	5	6
SVM	0.99	0.94	0.88	3	7	9

### Parametric analysis

**Figure 12:**
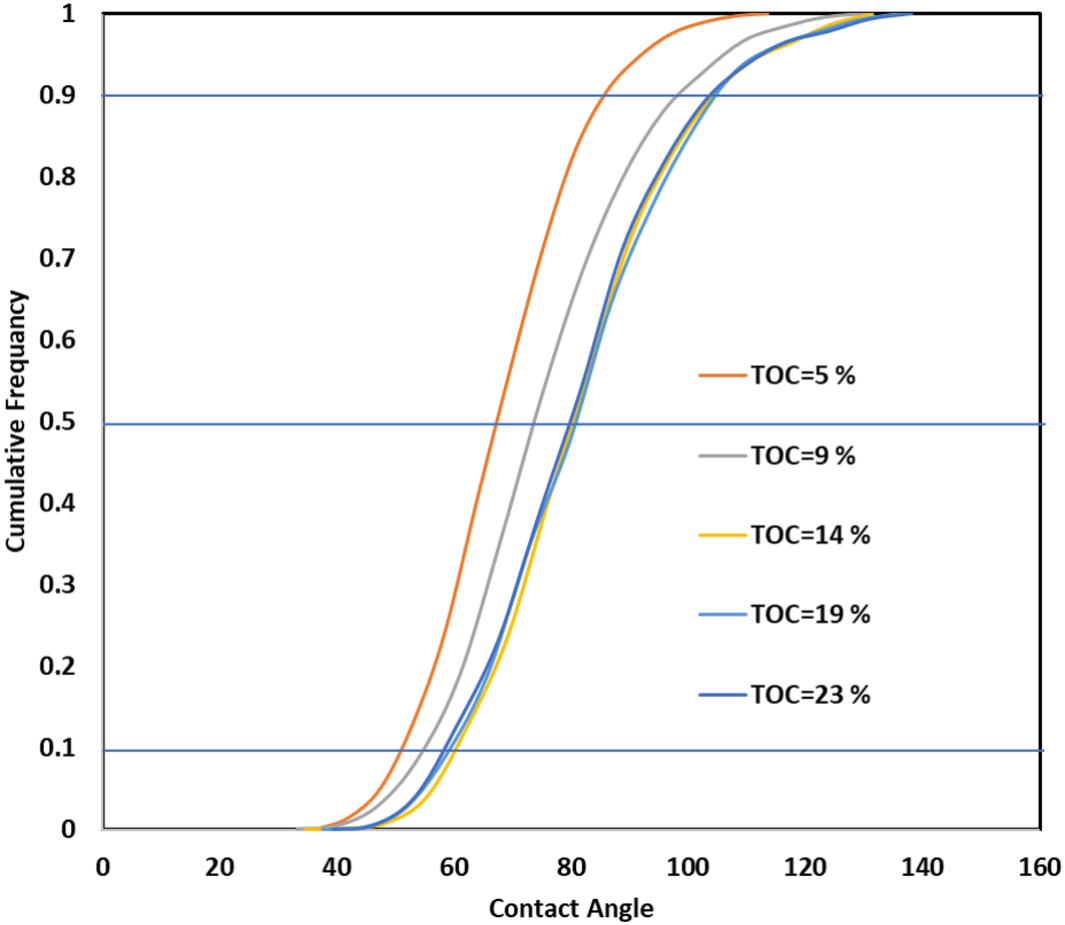
Cumulative frequency of different CA-values at different input properties for a certain TOC value.

The ANN model was used to investigate the sensitivity of CA-value to input parameters and TOC values. By generating 10,000 realizations, the CA-value was predicted as a function of input parameters within the ranges presented in Table 1 at specific TOC values. Figure 12 displays the cumulative frequency of the CA-value at different TOC values. For instance, when the TOC value is low (5%), the shale rock is mostly water-wet, regardless of the input parameter values. At a P (90) value, 90% of the samples will be water-wet at any conditions for low TOC values, and less than 10% of the samples will be CO_2_-wet with CA values higher than 90 degrees.

As the TOC value increases to 9%, the entire curve shifts to the right, indicating an increase in rock hydrophobicity. The findings revealed that for a shale sample with a TOC value of 9%, 25% of the shale samples will be CO_2_-wet. A similar trend was observed when the TOC value increased to 14%, where the shale surface becomes even more CO_2_-wet, and around 35% of the rock samples become CO_2_-wet. However, further increase in the TOC value did not change the distribution of the contact angle values, which suggests a high dependency of the wettability on the other input parameters.

**Figure 13:**
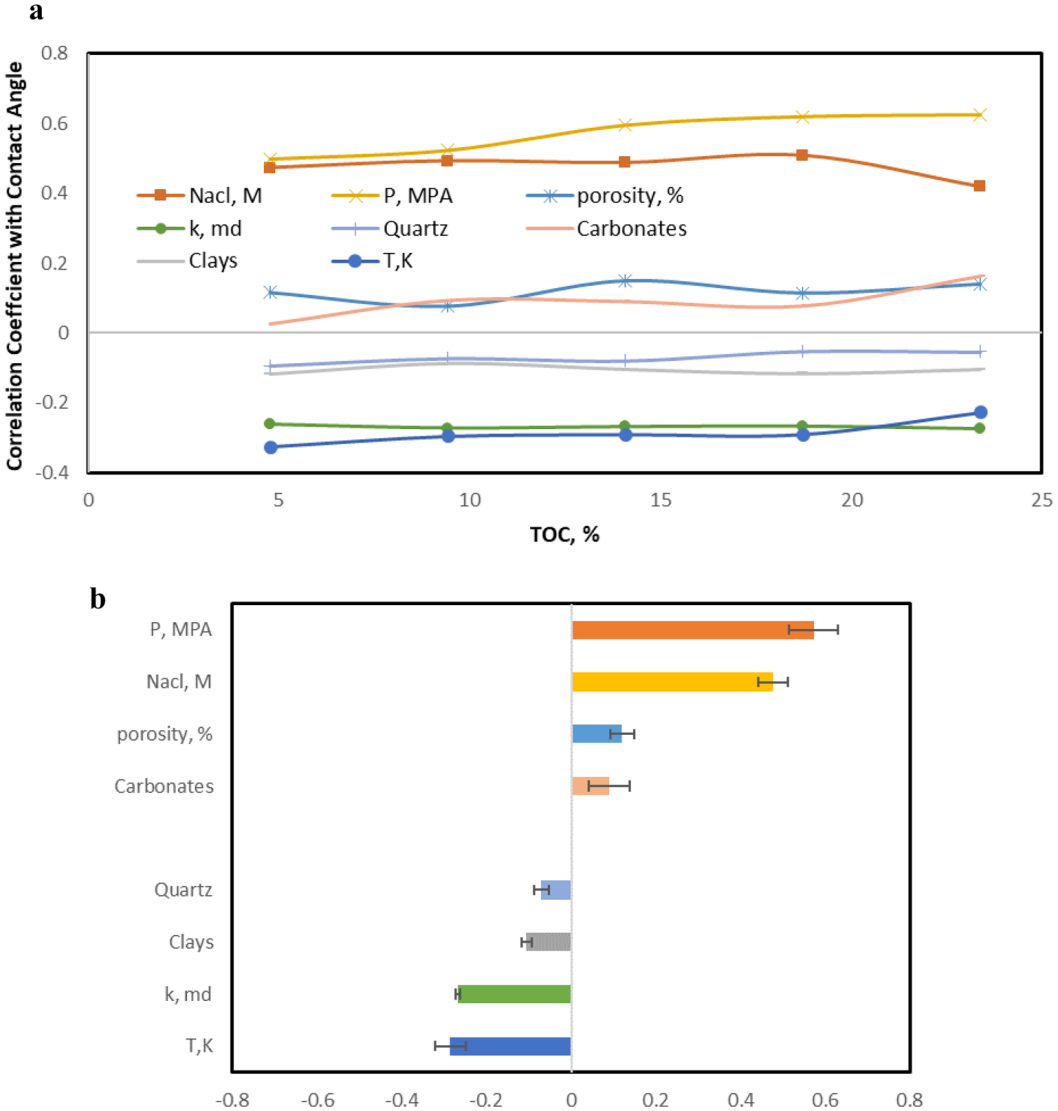
The CA-value sensitivity to the different input parameters. (**a**) The trend of R-value between the different parameters and contact angle for different TOC, (**b**) average R-values between the different parameters and contact angle.

R-values were calculated between the input parameters and the corresponding CA-value output values for each TOC value. Figure 13 shows the dependency of CA-value on various input parameters, which is independent of the TOC value. The R-values are almost constant for each parameter with increasing TOC values until a value of 9%. The R-value between the pressure and contact angle increased from 0.48 at low TOC values to 0.63 at high TOC values, confirming an increasing dependency of CA on pressure at high TOC values, consistent with the results shown in Fig. 12.

Figure 13b shows the average R-value between the input parameters and the contact angle at different TOC values. Operating pressure has the highest positive effect on the CA-values, followed by salt concentration in the brine solution. On the other hand, temperature has the most significant negative effect on the CA-values. The concentrations of clays and quartz display a negative relationship with the CA-value, indicating that the shale surface becomes more water-wet as their concentrations increase. In contrast, carbonates show a positive relationship with the CA-value, where the shale surface wettability turns less hydrophilic as the carbonate concentrations increase.

## Conclusions

This study utilized machine learning techniques, including ANN, ANFIS, and SVM, to predict the contact angle of shale formations under various operational conditions, eliminating the need for expensive and time-consuming experimental measurements. The results of this study are outlined below.

- The different ML models accurately predicted contact angle based on shale mineralogy and operating conditions.
- ANN model outperformed other ML models with R^2^ higher than 0.96 and RMSE less than 5 for training, testing and validation datasets.
- SVM model showed overfitting problem with R^2^ values decreased from 0.99 to 0.88 for the training dataset compared to the validation dataset.
- An empirical correlation was developed based on ANN model to predict contact angle without rerunning ML models with validation R^2^ of 0.96.
- Operating pressure had the most significant impact on shale wettability at constant TOC, and contact angle dependency on pressure increased at high TOC values.

## Data Availability

A summary of the data used in this study is included in the paper, and detailed data sample will be available upon request by contacting the corresponding Author (ahmed.ibrahim@kfupm.edu.sa).

## Abbreviations

ANN: Artificial neural networks
SVM: Support vector machines
ANFIS: Adaptive neuro fuzzy inference system
TOC: Total organic content
RMSE: Root mean square error
NMR: Nuclear magnetic resonance
CA: Contact angle
ML: Machine learning
R^2^: Coefficient of determination
Nacl, M: Molar brine concentration
K: Permeability
T: Operating temperature, K
P: Operating pressure, MPa
SS_E_: Summation of residuals squares
n: Number of data
$y_{i}$: Actual data values
$\hat{y}_{i}$: Predicted data values
$S_{YY}$: Summation of squares of data variation with respect to the data mean value
W_2i_ and b_2_: Weight and bias between the hidden layer and output layer
${\text{W}}_{{1{\text{i}},{\text{j}}}}$: Weights between the input layer and hidden layer
b_1i_: Optimized biases for the hidden layer neurons
N: Total number of neurons ()
